# Analysis of antiviral drug properties of thymidine kinase of herpes B virus using recombinant herpes simplex virus 1

**DOI:** 10.1128/spectrum.03091-23

**Published:** 2023-12-14

**Authors:** Phu Hoang Anh Nguyen, Shuetsu Fukushi, Souichi Yamada, Shizuko Harada, Tomoki Yoshikawa, Hitomi Kinoshita, Madoka Kawahara, Takuma Ogawa, Hideki Ebihara, Meng Ling Moi, Masayuki Saijo

**Affiliations:** 1 Department of Virology 1, National Institute of Infectious Diseases, Tokyo, Japan; 2 Department of Developmental Medical Sciences, Graduate School of Medicine, University of Tokyo, Tokyo, Japan; 3 Health and Welfare Bureau, Sapporo City, Hokkaido, Japan; Wayne State University, Detroit, Michigan, USA

**Keywords:** B virus, acyclovir, acyclovir resistant, ganciclovir, ganciclovir resistant, herpes simplex virus 1, thymidine kinases, DNA polymerase

## Abstract

Zoonotic infection by herpes B virus (BV) causes severe neurological disease, which in the absence of appropriate treatment can have a mortality rate of 80%. Long-term administration of acyclovir or ganciclovir is the recommended prophylactic treatment option for BV infections. However, the emergence of acyclovir- and/or ganciclovir-resistant BV may become a major problem. Here, we examined the ability of BV thymidine kinase (TK) from monkeys (mBVTK) and humans (hBVTK) to acquire resistance to acyclovir and ganciclovir. The sequences of mBVTK and hBVTK differ by two amino acids. We constructed and expressed four HSV-1-based recombinant viruses (HSV-1_mBVTK, HSV-1_hBVTK, HSV-1_VZVTK, and HSV_HSV-1TK) in which the HSV-1TK gene was deleted and the TK gene of monkey BV, human BV, varicella-zoster virus, and HSV-1 was inserted, respectively. We then tested the sensitivity of these recombinant viruses to acyclovir, ganciclovir, penciclovir, and brivudine. HSV-1_HSV-1TK and wild-type HSV-1 were more susceptible to ganciclovir, acyclovir, and penciclovir than HSV-1_VZVTK, HSV-1_hBVTK, and HSV-1_mBVTK. Both HSV-1_hBVTK and HSV-1_mBVTK exhibited similar sensitivity profiles against all tested drugs: both were susceptible to acyclovir, ganciclovir, and penciclovir but resistant to brivudine. In addition, we forced HSV-1_hBVTK to replicate under selective pressure from acyclovir or ganciclovir and then examined the ability of the BV TK gene to develop drug resistance. Investigation of the drug-resistant HSV-1_hBVTK gene revealed the absence of amino acid substitutions in BV TK, suggesting that the development of acyclovir- or ganciclovir-resistant BV through mutations in the TK gene is a rare occurrence in this experimental system.

**IMPORTANCE:**

Zoonotic infection of humans with herpes B virus (BV) causes severe neurological diseases. Acyclovir (ACV) and ganciclovir (GCV), most frequently used as anti-herpes drugs, are recommended for prophylaxis and therapy in human BV infection. In this study, we examined the property of BV thymidine kinase (TK) against anti-herpes drugs using a recombinant herpes simplex virus type 1 (HSV-1) carrying BV TK gene. We found that HSV-1 carrying BV TK was similarly sensitive to GCV as HSV-1 carrying varicella zoster virus TK. In addition, we demonstrated that BV TK was not mutated in the GCV- and ACV-resistant HSV-1 carrying BV TK, suggesting that ACV- or GCV-resistant BV might be rare during treatment with these antiviral drugs. These data can provide a new insight into the properties of BV TK in terms of the development of drug resistance.

## INTRODUCTION

*Simplexvirus macacinealpha1*, known as herpes B virus (BV), belongs to the same *Simplexvirus* genus, *Alphaherpesvirinae* subfamily, *and Orthoherpesviridae* family as *simplexvirus humanalpha1* (HSV-1) and *simplexvirus humanalpha2* (HSV-2) (1). Typically, BV infection of its healthy natural host (macaques) is asymptomatic, with the virus becoming latent in the dorsal root and trigeminal ganglia; however, it can periodically reactivate from latency and cause herpetic lesions (2). BV infection in humans was first reported in 1932; the mortality rate can reach approximately 80% if patients remain untreated (3 – 5).

In some cases, recovery from BV infection is possible without antiviral therapy (6); however, anti-herpesvirus drug treatment is highly recommended because BV disease is severe (4). Furthermore, long-term prophylactic use of anti-herpetic drugs is recommended due to the high frequency of reactivation. Acyclovir (ACV) and ganciclovir (GCV), which are drugs used to treat patients with HSV or VZV infections, are used for both prophylaxis and therapy in humans infected with BV (5, 7).

However, *in vitro* and *in vivo* studies of BV infection suggest that ACV might be less effective against BV than GCV; indeed, GCV is considered to be the most potent drug currently available for the treatment of patients with BV infections (8, 9). Moreover, although the protective efficacy of ACV and GCV has been demonstrated in some patients (10 – 12), there are many for whom anti-herpetic drug treatment is ineffective (13 – 15).

Thymidine kinase (TK), encoded by the UL23 gene of HSV-1, plays a crucial role in activating nucleoside analogs such as ACV, GCV, penciclovir (PCV), and brivudine (BVDU). Viral TK is necessary for the phosphorylation of these anti-herpetic drugs. Phosphorylation of ACV by viral TK in HSV-1-infected cells yields ACV-monophosphate (ACV-MP) (16 – 18), which is then phosphorylated by host cellular kinases and converted to the active triphosphate form (ACV-TP) *via* ACV diphosphate (ACV-DP). ACV-TP is incorporated into viral DNA by viral DNA polymerase (DNApol), which is encoded by the UL30 gene, thereby causing inhibition of viral DNA synthesis and viral replication (19, 20).

There is a possibility that the variation in the TK gene nucleotide sequence affects the sensitivity against antiviral drugs since the variable sensitivities among BV isolates to ACV and GCV were observed (21). Notably, PCV- and GCV-resistant BV has been isolated from a cynomolgus macaque; these resistant mutants carry a single nucleotide deletion in the BV TK gene (9, 22). Therefore, we must take into account the emergence of antiviral drug-resistant BV when treating patients with BV disease.

A recent study used an HSV-1 recombinant bacterial artificial chromosome (BAC) system to express varicella-zoster virus (VZV) TK instead of HSV-1 TK (23). It has been shown that, using the recombinant HSV-1, VZV TK is responsible for the reduced sensitivity against ACV. The result correlates with the fact that a high-dose regimen of ACV is required for the treatment of humans infected with VZV as compared with that with HSV-1 because of its relatively lower anti-VZV activity (24, 25). This recombinant HSV-1 system is useful for analyzing the properties of various/mutant TKs from other herpes viruses after exposure to anti-herpes drugs.

Here, we generated recombinant HSV-1_BVTKs in which the HSV-1 TK gene was deleted, and the TK gene of BV was inserted in the position between UL50 and UL51 of the HSV-1 genome. The BV TK genes from monkeys (mBVTK) and humans (hBVTK) were used; there were two amino acid differences between these two BV TK polypeptides. We then used these BV TK genes to construct two recombinant HSV-1 constructs, HSV-1_hBVTK and HSV-1_mBVTK, which express the hBVTK and mBVTK polypeptide, respectively. We then explored the effects of the two amino acid differences on drug sensitivity. We compared the inhibitory effects of GCV, ACV, PCV, and BVDU on replication of HSV-1_hBVTK and HSV-1_mBVTK with those on replication of a parental recombinant HSV-1_HSV-1TK and wild-type (WT) HSV-1. In addition, we placed HSV-1_hBVTK strains under selective pressure from ACV and GCV to generate drug-resistant strains to assess the contribution of the TK gene to the development of drug resistance.

## RESULTS

### Construction of recombinant pHSV-1_BVTK

**Fig 1:**
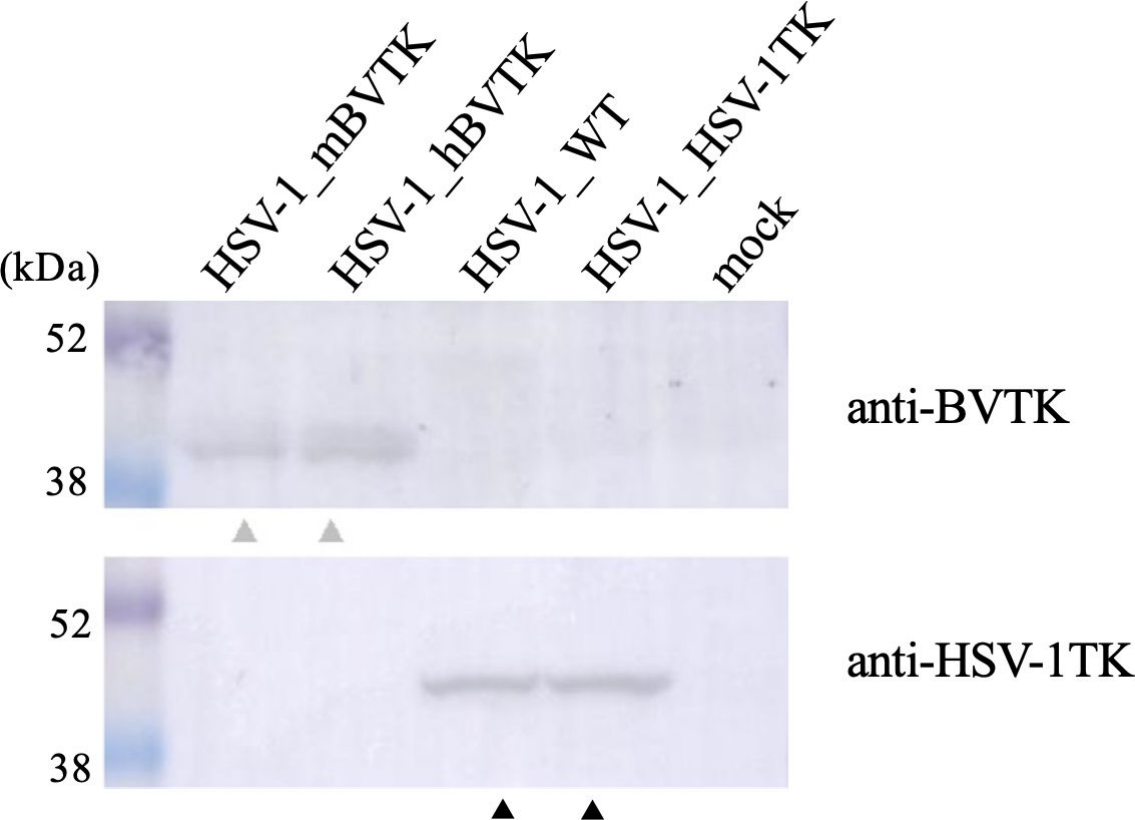
Expression of the viral TK polypeptides in Vero cells infected with HSV-1_hBVTK, HSV-1_mBVTK, HSV-1_HSV-1TK, HSV-1_WT, and mock at a multiplicity of infection of 3 PFU, as assessed with Western blot analysis. The antibody raised against viral TK is shown on the right. Vero cells were infected with the indicated HSV-1 recombinants or mock. Gray and black arrows denote BVTK and HSV-1TK protein, respectively.

HSV-1_hBVTK and HSV-1_mBVTK were generated using the HSV-1 BAC system. Expression of TK proteins, reconstituted in Vero cells infected with each recombinant HSV-1, was confirmed by Western blot analysis using anti-BV TK and anti-HSV-1 TK serum. Anti-BV TK reacted with a band corresponding to the size (approximately 42 kDa) of the BV TK protein in cells infected with HSV-1_hBVTK and HSV-1_mBVTK, but not in those infected with HSV-1_HSV-1TK and HSV-1_WT. By contrast, anti-HSV-1 TK reacted with a band corresponding to the size (approximately 42 kDa) of the HSV-1 TK protein in cells infected with HSV-1_HSV-1TK and HSV-1_WT, but not in those infected with HSV-1_hBVTK and HSV-1_mBVTK (Fig. 1). It should be noted that the expression level of HSV-1 TK in HSV-1_HSV-1TK (driven from CMV promoter) was the same as that of HSV-1_WT (driven from native promoter).

**Fig 2:**
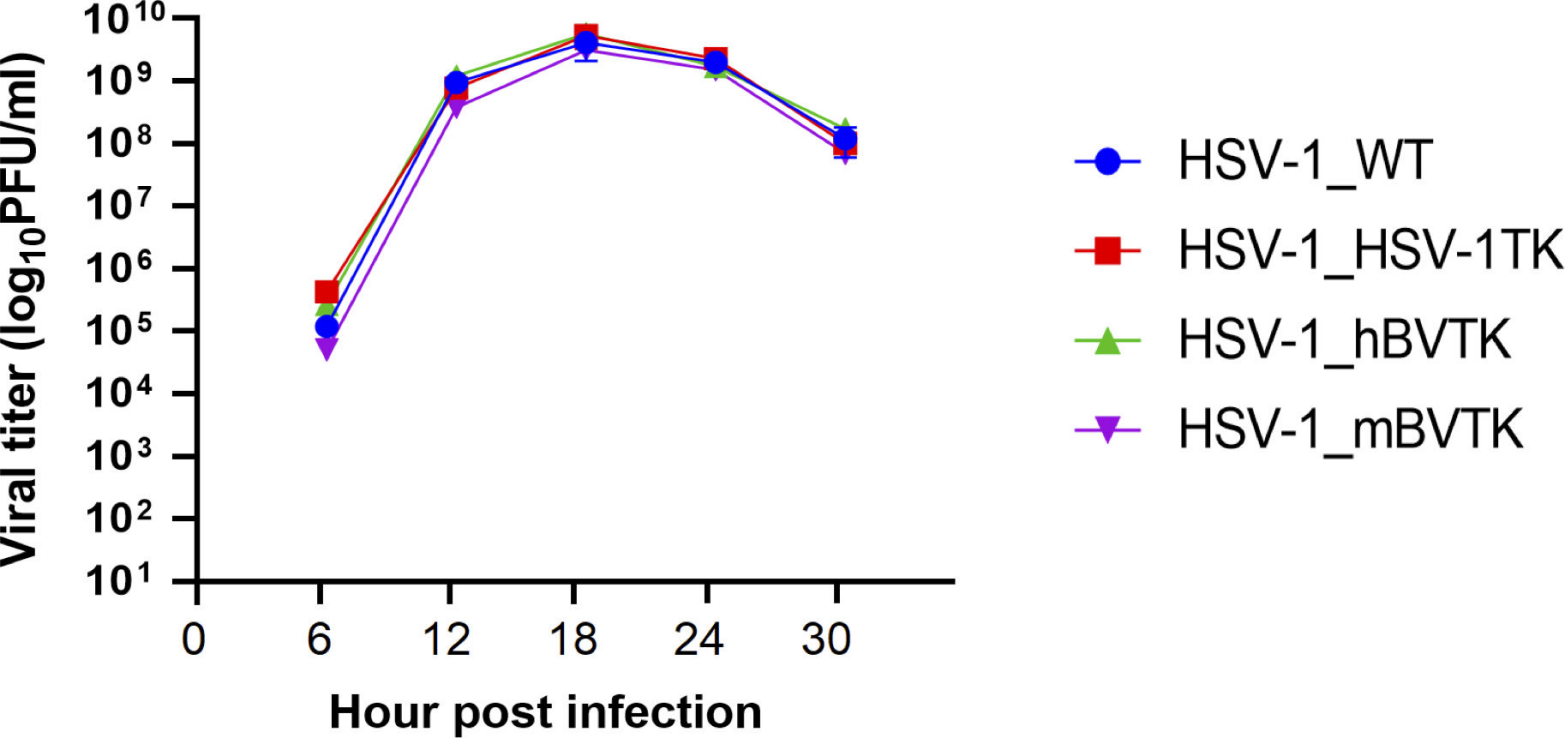
The growth properties of HSV-1_WT, HSV-1_HSV-1TK, HSV-1_hBVTK, and HSV-1_mBVTK. Vero cells were infected with each virus at a multiplicity of infection of 2 PFU per cell, harvested at the designed time points, and titrated in a plaque assay. The experiments were repeated twice independently. Data are presented as the mean ± standard deviation of the two repeat experiments.

Vero cells were inoculated with each recombinant HSV-1 at a multiplicity of 2 PFU per cell, and viral titers were measured over time to examine how the reconstituted TK gene in HSV-1 recombinant viruses affected viral replication. All of the recombinant viruses tested displayed equivalent viral replication kinetics, confirming that the reconstituted TK genes did not affect replication capacity in Vero cells (Fig. 2).

### Susceptibility of recombinant HSV-1 viruses to anti-herpes drugs

**Fig 3:**
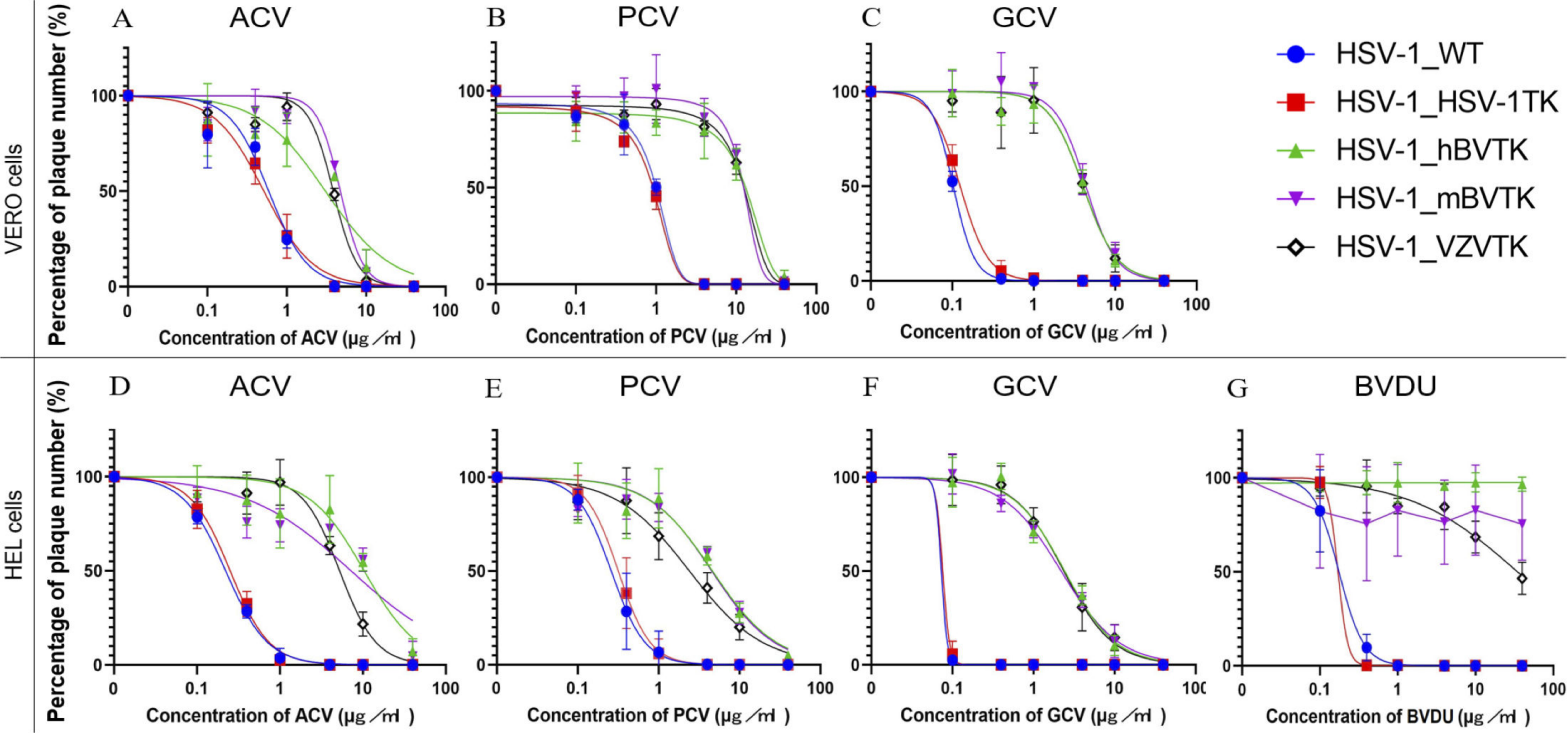
The sensitivity of the recombinant virus to ACV, PCV, GCV, and BVDU was determined in a plaque reduction assay on Vero cells (A, B, and C, upper panel) and HEL cells (D, E, F, and G, lower panel). The replication curves for each virus in the presence of the designated concentration of antiviral drug are shown. The number of plaques formed by each virus at the lowest drug concentration was almost identical to that formed by the mock (control). Each concentration was tested in triplicate, and the experiments were repeated independently three times. Data are presented as the mean ± standard deviation of the three repeat experiments.

The anti-herpes virus activity of compounds depends on the cell type infected (26). It has been shown that antiviral activities of compounds are different between Vero and HEL cells, due to the difference in the size of the thymidine, thymidine phosphate pools, and thymidine synthetase activity (26, 27). Therefore, Vero and HEL cells were used to test the sensitivity of recombinant HSV-1s to ACV, PCV, and GCV (Fig. 3). The sensitivity to BVDU was tested only in infected HEL cells (Fig. 3) because it had already been shown that BVDU showed weak/absent activity against alpha herpesviruses in Vero cells, whereas activity was high if tested in infected HEL cells (28). The inhibitory concentration of each drug required to reduce viral plaque formation by 50% (IC_50_) is shown in Table 1.

**TABLE 1:** Susceptibility profiles of recombinant HSV-1 viruses to the TK-associated drugs tested in Vero cells and those tested in HEL cells

Viruses	IC_50_ (mean ± SD µg/mL)								
	Vero cells					HEL cells			
	ACV	PCV	GCV			ACV	PCV	GCV	BVDU
HSV-1_WT	0.5 ± 0.1^*a*^	1.1 ± 0.2^*a*^	0.1 ± 0^*a*^			0.2 ± 0.1^*a*^	0.3 ± 0.1^*a*^	0.01 ± 0^*a*^	0.2 ± 0.06^*a*^
HSV-1_HSV1TK	0.6 ± 0.1^*a*^	0.9 ± 0.2^*a*^	0.1 ± 0.02^*a*^			0.2 ± 0.1^*a*^	0.3 ± 0.1^*a*^	0.01 ± 0^*a*^	0.2 ± 0.01^*a*^
HSV-1_hBVTK	4.7 ± 0.3^*b*^	13 ± 2.3^*c*^	4.1 ± 0.6^*c*^			12 ± 0.7^*b*^	5.1 ± 0.7^*b*^	2.4 ± 0.1^*c*^	>40^*c*^
HSV-1_mBVTK	5.1 ± 0.3^*b*^	14 ± 1.2^*c*^	4.4 ± 0.7^*c*^			12 ± 1.7^*b*^	5.3 ± 0.3^*b*^	2.3 ± 0.2^*c*^	>40^*c*^
HSV-1_VZVTK	3.8 ± 0.3	14 ± 1.8	4.0 ± 0.6			5.3 ± 0.2	2.2 ± 0.5	2.1 ± 0.03	>40

^*a*^ Significantly different from HSV-1_hBVTK, HSV-1_mBVTK, and HSV-1_VZVTK (*P* < 0.01).

^*b*^ Significantly different from HSV-1_VZVTK (*P*<0.01). No significant difference between HSV-1_hBVTK and HSV-1_mBVTK.

^*c*^ Not significant from HSV-1_VZVTK. No significant difference between HSV-1_hBVTK and HSV-1_mBVTK.

Both HSV-1_WT and HSV-1_HSV-1TK in Vero cells showed high sensitivity to ACV, whereas HSV-1_VZVTK showed reduced sensitivity, as reported previously (23). The sensitivity of HSV-1_WT and HSV-1_HSV-1TK to ACV, PCV, and GCV was higher than those of HSV-1_hBVTK and HSV-1_mBVTK (Fig. 3). The results agreed with the previous experimental observations that ACV and GCV were less efficient substrate for recombinant BV TK protein (29) and that BV was less sensitive to ACV and GCV than HSV-1 (21). Furthermore, when sensitivity was tested in Vero cells, the sensitivity profiles of HSV-1_hBVTK and HSV-1_mBVTK were similar to those of HSV-1_VZVTK (Table 1). The sensitivity pattern of these recombinant viruses to antiviral compounds in Vero cells was almost the same as that observed in HEL cells (Fig. 3). There was no significant difference in the sensitivity to these compounds between HSV-1_hBVTK and HSV-1_mBVTK (Table 1).

The IC_50_ values of ACV and PCV against HSV-1_hBVTK and HSV-1_mBVTK were twofold higher than those against HSV-1_VZVTK in HEL cells but not in Vero cells (Table 1). The discrepancy might be reflected by the cell-dependent anti-herpes drug property of BV TK, as it is known that the anti-herpesvirus activity of compounds varies between Vero and HEL cells (26, 27), although a more detailed analysis of TK activity both in HEL and Vero cells when infected with HSV-1_BVTKs is required to prove this possibility. By contrast, the IC_50_ values of GCV against HSV-1_hBVTK and HSV-1_mBVTK were comparable with that of HSV-1_VZVTK both in Vero and HEL cells (Table 1), indicating that HSV-1_hBVTK and HSV-1_mBVTK were less sensitive to ACV and GCV than HSV-1_HSV-1TK but were as sensitive to GCV as HSV-1_VZVTK.

The IC_50_ of PCV for HSV-1_hBVTK and HSV-1_mBVTK was comparable with, or higher than, that of HSV-1_VZVTK both in Vero and HEL cells but was more than 10-fold higher than that for HSV-1_HSV-1TK. In addition, BVDU did not inhibit the replication of HSV-1_hBVTK or HSV-1_mBVTK as reported previously (29). There was a statistically significant difference in the IC_50_ values of these compounds between the susceptible group (HSV-1_WT and HSV-1_HSV-1TK), and the less sensitive group (HSV-1_VZVTK, HSV-1_hBVTK, and HSV-1_mBVTK) with the *P* value of < 0.01.

### Characterization of ACV- and GCV-resistant HSV-1_hBVTK

**Fig 4:**
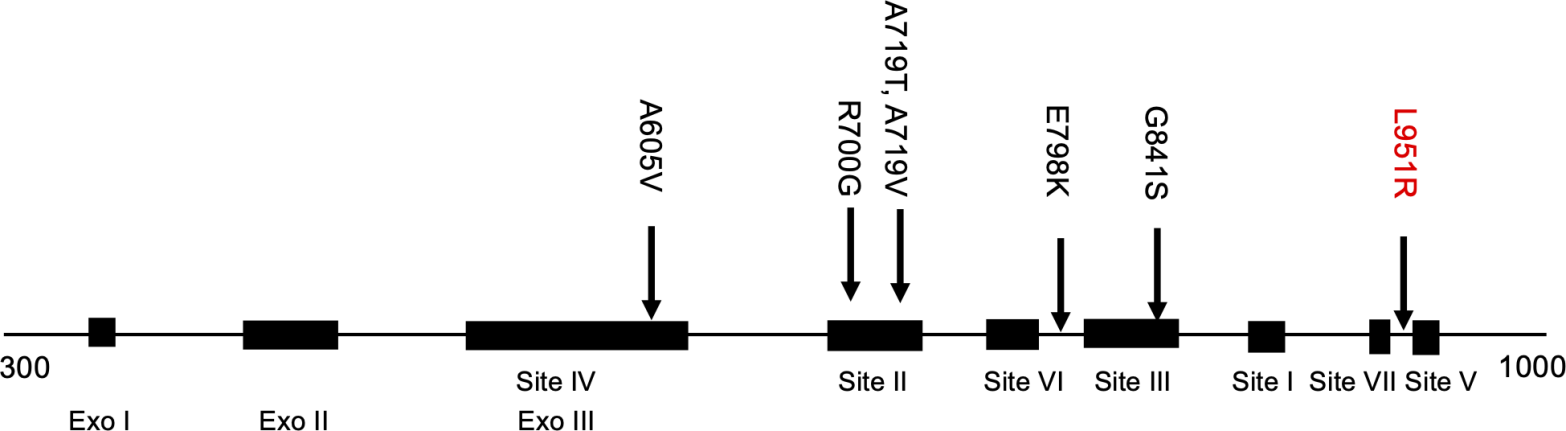
Schematic representation of amino acid substitutions in UL30. The conserved regions (sites I–VII) and functional domains (exo I–III) are presented in black boxes. The amino acid substitutions reported to confer ACVr (23, 26), as well as that identified in this study (L951R), are denoted by black and red letters, respectively.

A serial passage of HSV-1_hBVTK was performed in the presence of increasing concentrations of ACV or GCV to elucidate the mechanism by which recombinant HSV-1 expressing BVTK acquired drug resistance. In all, 15 ACV-resistant (ACVr) HSV-1_hBVTK clones were obtained. In these ACVr clones, the IC_50_ values of ACV increased by more than 10-fold in comparison to the parental HSV-1_hBVTK (Table 2). Sequence analysis indicated that none of the ACVr HSV-1_hBVTK clones harbored mutations in the BVTK gene when compared with the parental HSV-1_hBVTK clone but they did acquire a single nucleotide mutation in the HSV-1 DNA polymerase (DNApol) gene, resulting in a single amino acid change either in conserved regions (12 clones) or non-conserved regions (three clones) (Fig. 4). The location of these mutations was almost the similar to those of mutations detected in the ACVr HSV-1_VZVTK reported previously (23, 30). The exception was the amino acid mutation of L951R (Fig. 4).

**TABLE 2:** IC_50_ values and amino acid substitutions in the ACVr or GCVr HSV-1_hBVTK clones

Viruses	Clones	IC_50_ (mean ± SD µg/mL)		Amino acid substitutions	
		GCV	ACV	TK	DNApol
HSV-1_WT		0.03	0.5 ± 0.1	n. t.^*a*^	n. t.
HSV-1_hBVTK		3.9 ± 0.6	4.2 ± 0.2	n. d.^*b*^	n. d.
HSV-1 TAR^*c*^		9.8 ± 1.9	21 ± 2.7	n. t.	n. t.
ACVr HSV-1_hBVTK^*d*^	Clone 1	n. t.	53 ± 11	n. d.	L951R
	Clone 2	n. t.	76 ± 5	n. d.	G841S
	Clone 3	n. t.	>100	n. d.	G841S
	Clone 4	n. t.	>100	n. d.	R700G
	Clone 5	n. t.	>100	n. d.	E798K
	Clone 6	n. t.	>100	n. d.	E798K
	Clone 7	n. t.	87 ± 12	n. d.	A605V
	Clone 8	n. t.	>100	n. d.	A719T
	Clone 9	n. t.	94 ± 5	n. d.	A719T
	Clone 10	n. t.	>100	n. d.	A719V
	Clone 11	n. t.	>100	n. d.	A719V
	Clone 12	n. t.	>100	n. d.	A719V
	Clone 13	n. t.	>100	n. d.	A719V
	Clone 14	n. t.	>100	n. d.	A719V
	Clone 15	n. t.	78 ± 11	n. d.	A719V
GCVr HSV-1_hBVTK^*e*^	Clone 1	28 ± 2.2	17 ± 2.4	n. d.	n. d.
	Clone 2	35 ± 2.9	23 ± 2.9	n. d.	n. d.
	Clone 3	18 ± 1.3	12 ± 2.4	n. d.	n. d.
	Clone 4	16 ± 3.9	16 ± 2.6	n. d.	n. d.
	Clone 5	25 ± 2.9	19 ± 2.9	n. d.	n. d.
	Clone 6	19 ± 2.2	17 ± 5.2	n. d.	n. d.
	Clone 7	11 ± 0.7	5.9 ± 1.2	n. d.	n. d.
	Clone 8	17 ± 4.0	25 ± 3.1	n. d.	n. d.
	Clone 9	39 ± 7.8	16 ± 1.0	n. d.	n. d.
	Clone 10	15 ± 2.4	6.5 ± 1.0	n. d.	n. d.
	Clone 11	29 ± 1.6	24 ± 5.3	n. d.	n. d.
	Clone 12	49 ± 2.0	33 ± 2.0	n. d.	n. d.

^*a*^ Not tested.

^*b*^ Not detected.

^*c*^ HSV-1 TAR isolated from a child with Wiskott-Aldrich syndrome resistant to ACV due to a single nucleoside frameshift mutation in the viral TK gene.

^*d*^ Drug-resistant HSV-1_hBVTK clones generated in the presence of ACV.

^*e*^ Drug-resistant HSV-1_hBVTK clones generated in the presence of GCV.

In all, 12 GCVr HSV-1_hBVTK clones were obtained after serial passage in the presence of GCV. In these GCVr clones, the IC_50_ values of GCV increased 2.8- to 12.5-fold in comparison to the parental HSV-1_hBVTK (Table 2). Surprisingly, sequence analysis indicated that none harbored mutations neither the BV TK gene nor HSV-1 DNApol gene. We found neither mixture nor frameshift mutation on the TK/DNA pol genes. Since none of the GCVr HSV-1_hBVTK clones had mutations in the BV TK genes or DNApol genes, we examined their sensitivity to ACV. The GCVr HSV-1_hBVTK clones were 1.4–7.7 times less susceptible to ACV than the parental HSV-1_hBVTK, indicating that these clones had acquired resistance to ACV (Table 2).

## DISCUSSION

Two Japanese patients, who had been diagnosed with non-infectious neurological diseases, were diagnosed retrospectively as having BV-mediated disease (31). Thus, the differential diagnosis of neurological disease and BV disease is very difficult, but important because there is a specific antiviral drug treatment strategy for patients with BV disease. Administration for patients with BV disease with antiviral agents such as GCV, PCV, and ACV should be initiated as early as possible from disease onset to make the outcome of the patients better. Furthermore, patients who survived and/or recovered from BV disease should be administered for prophylaxis with the antiherpetic drug for the long term to prevent BV reactivation from latency. Therefore, the susceptibility and/or resistance of BV to antiviral drugs is a major concern that should be addressed.

Here, we found that the antiviral sensitivity profile of HSV-1_hBVTK was similar to that of HSV-1_mBVTK. These data suggest that the difference in the amino acid sequence of BV TK by two amino acid residues between hBVTK and mBVTK did not affect the drug sensitivity profiles.

We used a recombinant HSV-1 carrying the BV TK gene to examine the effects of anti-herpes drugs on the viral TK. HSV-1_hBVTK and HSV-1_mBVTK were as sensitive to GCV as HSV-1_VZVTK but less susceptible to ACV, GCV, and PCV than HSV-1_HSV-1TK and HSV-1_WT. The recombinant viruses harboring various viral TK genes, all of which had the same HSV-1 genome backbone, exhibited varying degrees of susceptibility to ACV, indicating that susceptibility of BV to TK-associated anti-herpesvirus drugs depends solely on the nature of BV TK itself. This is supported by earlier research by Krug et al., who showed that BV was less sensitive to ACV and GCV than HSV-1, possibly due to the variations in the nature of each TK protein (21). Similarly, the properties of VZV TK are responsible for the reduced sensitivity of the recombinant HSV-1 carrying the VZV TK gene to ACV (23). An earlier study has also demonstrated that antiviral drug sensitivities of recombinant HSV carrying VZV TK resemble VZV rather than HSV (32).

Interestingly, BVDU had little inhibitory effect on HSV-1_VZVTK in HEL cells (Fig. 3; Table 1). It is an unexpected result since BVDU has been used for the treatment of patients with VZV infection. Although the mechanism for this little inhibitory effect is unclear, the effect might probably be due to a reduced phosphorylation activity of VZV TK expressed in HEL cells against BVDU or BVDU-monophosphate. Consistent with the findings of this study, it has been shown that anti-VZV activity of BVDU in HEL cells is 10-fold lower than that in Vero cells (27). By contrast, BVDU exhibits lower anti-HSV-1 potencies in Vero cells than in HEL cells (33). The difference in anti-viral potencies in Vero and HEL cells may be attributed to the difference in the levels of thymidine kinase activity and/or thymidine pool in infected cells (26, 32, 33). The difference in experiment settings (e.g., preparation of cells, compounds, viruses) might also affect anti-viral potencies.

In addition, the potency of an antiviral drug is dependent on its ability to act as a substrate for BV TK. ACV was less effective against BV than the other TK-associated nucleoside analogs. The reduced ACV-phosphorylation activity of BV TK correlates with its reduced efficacy against BV (29). Taken together, the data presented herein suggest that differences in the susceptibility of HSV-1 and BV to anti-herpesvirus drugs depend on the characteristics in terms of the phosphorylation activities of the TK polypeptide of each virus.

Several clinical studies report the efficacy of GCV as a treatment for VZV infection in humans, despite the lack of *in vitro* and *in vivo* data linking suppression of VZV to GCV (34, 35). The Infectious Diseases Society of America (IDSA) recommends GCV as a substitute therapy for VZV-associated encephalitis (36). The results of our study provide important insight into the sensitivity of BV, which causes encephalitis in patients, to TK-associated anti-herpesvirus drugs, as well as the ability of GCV to inhibit BV replication to a level comparable with that of VZV.

Sequence analysis of 15 ACVr HSV-1_BVTK clones revealed that HSV-1_hBVTK acquired ACV resistance not through mutations in the BV TK gene but solely through mutations in the HSV-1 DNApol gene. These findings demonstrate that, like VZV TK, BV TK is unlikely to accumulate the mutations required for ACV resistance (23). Similar results have been obtained by previous study (23). ACV-resistant HSV-1_VZVTK harbors mutation on the DNApol gene but not on the TK gene. However, all ACV-resistant HSV-1_HSV-1TK harbor mutations on HSV-1 TK (23). The mechanism underlying the difference in the mutated genes associated with ACV resistance might be due to the characteristics of viral TKs. Mutation on DNApol in the ACVr HSV-1_BVTK clones might result from the selective pressure of ACV on DNA polymerase rather than the BV TK which has low affinity to ACV (29).

GCV is recommended for patients with BV disease based on the sensitivity of BV to this drug. Because of the increased toxicity of GCV compared with ACV, high doses and long-term administration come with an increased risk of side effects. Moreover, the issue of GCV resistance is a concern for patients with BV disease. In this study, we generated 12 GCVr HSV-1_hBVTK clones. As was the case for ACVr HSV_hBVTK clones, no GCVr HSV-1_hBVTK harboring mutations in the BV TK gene of HSV-1_hBVTK were generated by serial passage of the virus under selection pressure from GCV. Western blot analysis of the BV TK protein indicated that a polypeptide corresponding to the intact protein was expressed in Vero cells infected with each GCVr HSV-1_hBVTK clone (data not shown). This suggests that BV TK does not have the capacity to acquire GCV resistance-conferring mutations in the recombinant HSV-1 backbone. There are no hot spots in the BV TK genes that confer mutations associated not only with ACV resistance but also with GCV resistance.

There was a clear difference in the occurrence of drug-resistance-associated mutations in the HSV-1 DNApol gene between ACVr HSV-1_hBVTK clones and the GCVr HSV-1_hBVTK clones. All ACVr HSV-1_hBVTK clones harbored mutations in the DNApol gene, which might confer resistance to ACV. By contrast, none of the GCVr HSV-1_hBVTK clones had mutations in both the BV TK and HSV-1 DNApol genes; however, these clones showed cross-resistance to ACV. A clone with similar properties was obtained in a previous study (23), in which ACVr HSV-1_VZVTK was generated in the same way as HSV-1_hBVTK in the present study. One of 31 ACVr HSV-1_VZVTK harbored no mutations in either the TK or DNApol genes when compared with the original HSV-1_VZVTK (23). To the best of our knowledge, the mechanism underlying the acquisition of ACV or GCV resistance has always been associated with mutations in either the TK or DNApol genes. However, there may be other mechanism(s), other than acquiring drug resistance conferring mutations in the TK and DNApol genes. A plausible hypothesis is that the viral DNA replication complex might contribute to this phenomenon. The core replication machinery of HSV-1 is composed of at least seven viral proteins including the origin binding protein (UL9), single-stranded DNA binding protein (UL29), helicase-primase complex (UL5, UL8, and UL52), DNA polymerase (UL30), and the processivity factor (UL42) (37). It is assumed that amino acid substitution(s) on DNA replication machinery proteins, other than UL30, also influences the catalytic reactions of ACV- or GCV-triphosphate during viral DNA synthesis. These proteins represent promising candidates for further investigation into the mechanisms underlying the development of ACV and GCV resistance. An alternative possibility is that a more complex phosphorylation pathway in which both ACV and GCV might serve as substrates not only for HSV-1 TK but also for the protein kinases (PK) such as HSV-1 UL13. The phosphorylation of GCV may depend not solely on TK but also on the PK (38). The UL47 of VZV and UL97 of CMV encodes PK capable of phosphorylating GCV (38). In addition, the Epstein-Barr virus (EBV) encodes a PK BGLF4, which has been demonstrated to coordinate the phosphorylation of both GCV and ACV within EBV-infected cells (39). The HSV-1 UL13 is one of the serine/threonine PK which shares functional features similar to VZV-UL47, CMV-UL97, and EBV-BGLF4. Specifically, they could phosphorylate both viral and cellular proteins (39, 40). This raises an intriguing question about whether the use of GCV or ACV could apply selective pressure, potentially leading to modifications not just within HSV-1 TK but also within the HSV-1 PK. Further study is needed for elucidation of the mechanism. Given the limitations of Sanger sequencing in sensitivity in detecting minor genetic variants, conducting further research analysis, including whole-genome sequencing of GCVr HSV-1_hBVTK clones is essential to clarify this matter. Also, we do not know whether the as-yet-unknown mechanism is specific to recombinant HSV-1, or is also common to wild-type alpha herpesviruses.

This study presents valuable advantages, offering a BSL-2 compatible approach using recombinant HSV-1_BVTKs, obviating the need for high-security BSL-4 facilities in antiviral drug assessment. This study confirmed the susceptibilities of BV to TK-associated antiherpetic compounds by developing recombinant HSV-1. This system could be useful for further analysis of BV TK properties.

## MATERIALS AND METHODS

### Cells

African green monkey kidney-derived cell line (Vero cells, CCL-81), purchased from American Type Culture Collection (Manassas, MA), human embryonic lung fibroblast (HEL) cells, and African green monkey kidney fibroblast-like (COS-7) cells (33) were used to propagate viruses, test the susceptibility to antivirals, and reconstitute recombinant viruses. Insect Sf9 cells and Tn5 cells, derived from *Spodoptera frugiperda* and *Trichoplusia ni*, respectively, were used to construct BV TK protein-expressing recombinant baculovirus and for the production of recombinant BV TK protein, respectively. These cells were maintained as described previously (41, 42).

### Plasmids

The plasmid, pEP-KanS_hBVTK, consisted of a pEP-KanS vector containing a kanamycin resistance cassette and the DNA fragment of the hBVTK gene (GenBank no. LC764823) originating from a Japanese patient with BV disease (31), was generated as described previously (23, 43).

The hBVTK gene sequence differed from that of the mBVTK gene, originating from the laboratory rhesus macaque E2490 strain (GenBank no. AF533768.1). The former harbors two synonymous nucleotide differences (C279T and C681T) and two non-synonymous nucleotide differences (G764C and G982A), resulting in amino acid substitutions G255A and E328K. The plasmid pEP-KanS_mBVTK consists of a pEP-KanS vector containing the DNA fragment of the mBVTK gene, was also generated by introducing amino acid substitutions A255G and K328E into the pEP-KanS_hBVTK by PCR primer-based mutagenesis.

A DNA fragment containing the 50 nucleotides of the 3′-end of the CMV promoter, the full-length hBVTK gene and poly A tail, and a 40-nucleotide sequence of UL51 flanked by *BamHI* restriction sites, was amplified from pEP-KanS_hBVTK by PCR. This DNA fragment was inserted into the vector pAmp-9mScarlet-I (44) to yield pAmp-9mScarlet-I_hBVTK (Fig. S1). Transfer DNA, containing the 50 nucleotides of the 3′-end of the CMV promoter, an *I-Sce*I site, an ampicillin resistance cassette, the mScarlet-I gene, the 50 nucleotides of the 3′-end of CMV promoter, hBVTK gene and poly(A) region, and a 50 base pair sequence of the HSV-1 UL51 gene was amplified from pAmp-9mScarlet-I_hBVTK by PCR (Fig. S1). The expected nucleotide sequences of the CMV promoter region and the BV TK gene in all of the constructed plasmids were confirmed by Sanger DNA sequencing. The transfer DNA fragment was then used to construct recombinant HSV-1_hBVTK by two-step Red-mediated recombination, as described below. The mBVTK transfer DNA used to construct recombinant HSV-1_mBVTK was generated in the same way as that used to construct HSV-1_hBVTK.

The hBVTK gene fragment was inserted into the pAcYM1 transfer vector (42), resulting in transfer plasmid pAcYM1_hBVTK, which was then used to generate hBVTK-expressing recombinant baculovirus.

### Recombinant viruses

Recombinant HSV-1_WT was derived from pYEbac102-derived HSV-1 strain F. Recombinant HSV-1_VZVTK, which expresses the TK of VZV under the control of the CMV promoter, and from which the HSV-1 TK gene was deleted, was also used (23). Recombinant HSV-1_HSV-1TK, which expresses the TK of HSV-1, was generated by replacing the VZV TK gene of HSV-1_VZVTK with that of HSV-1 TK. These three recombinant viruses were developed previously (23). HSV-1 TAR, which acquired resistance to ACV through a frameshift mutation in the viral TK gene, was used as a drug-resistant control virus (45, 46). Recombinant HSV-1_HSV-1TK was used as a parental virus to construct recombinant HSV-1_BVTKs.

Recombinant HSV-1_hBVTK and HSV-1_mBVTK, which express hBVTK and mBVTK, respectively, under the control of the CMV promoter, and from which the HSV-1 TK gene was deleted, were constructed by Red-mediated recombination as described previously (23, 47). Reconstitution and propagation of recombinant HSV-1_WT, HSV-1_hBVTK, HSV-1_mBVTK, HSV-1_HSV-1TK, and HSV-1_VZVTK were performed as described previously (23). The infectious titers (PFU) of the recombinant viruses were measured in a plaque assay using Vero cells.

### Expression of recombinant hBVTK protein

The hBVTK-expressing recombinant baculovirus, designated as Ac_hBVTK, was generated by transfecting insect Sf9 cells with the transfer plasmid pAcYM1_hBVTK mixed with BestBac linearized baculovirus DNA (Expression systems, Davis, CA) (42). The Tn5 cells were infected with Ac_hBVTK for expression of recombinant hBVTK. Purification of the recombinant hBVTK protein was performed using the His-Bind Buffer Kit (Merck KGaA, Darmstadt, Germany).

### Anti-serum against TK

Three 11-week-old female BALB/c mice (SLC Japan, Kurume, Japan) were immunized with purified recombinant hBVTK; blood was collected after the third immunization, and serum (anti-BVTK serum) was collected and stored at −20°C. Anti-HSV-1 TK rabbit serum was also used (46).

### Compounds

ACV, PCV, GCV, and BVDU were purchased from the Tokyo Chemical Industry (Tokyo, Japan).

### Susceptibility of recombinant HSV-1 to anti-herpes compounds

The IC_50_ values of the anti-herpetic drugs (ACV, GCV, PCV, and BVDU) for HSV-1_hBVTK, HSV-1_mBVTK, HSV-1_WT, HSV-1_HSV-1TK, and HSV-1_VZVTK were determined in the plaque reduction assay as reported previously (41). Briefly, approximately 50 PFU of each virus was absorbed onto a Vero cell monolayer at 37°C for 1 h. The inoculum was then removed. Virus-inoculated Vero cells were cultured in Dulbecco’s Modified Eagle’s Medium containing 2% calf serum, designated concentrations of anti-herpetic drugs, and gamma-globulin (vol/vol 100:2). After 3 days of incubation at 37°C/5% CO_2_, virus-forming plaques were counted. The drug concentrations in the plaque reduction assay of each anti-herpetic drug tested were 0, 0.1, 0.4, 1, 4, 10, and 40 µg/mL. Each concentration was tested in triplicate, and each experiment was independently repeated three times. The same method was used to test the susceptibility of the recombinant viruses to anti-herpetic compounds after infection of HEL cells, except that cells were cultured in the Minimum Essential Medium containing 2% fetal bovine serum.

### Generation of ACV- and GCV-resistant HSV-1_hBVTK mutants

Drug-resistant HSV-1_hBVTK strains were generated as described previously (23, 48, 49). Briefly, Vero cells were infected with each plaque-purified HSV-1_hBVTK clone at an MOI of 0.1 and were then serially passaged on Vero cells in the presence of increasing concentrations of either ACV or GCV (1, 4, 10, 20, 40, 80, and 160 µg/mL). Each clone that replicated at the concentration of 160 µg/mL was obtained by plaque purification in a medium containing 160 µg/mL of the corresponding compound. The plaque-purified clones were propagated to generate virus stocks prior to the extraction of viral DNA.

### Determination of nucleotide sequence of the CMV promoter, BV TK, and HSV-1_DNApol genes

DNA fragments corresponding to the CMV promoter region, BV TK gene, and HSV-1 DNApol gene of drug-resistant clones were amplified by PCR, and their nucleotide sequences were determined by Sanger sequencing using BigDye Terminator v3.1 Cycle Sequencing Kit (Thermo Fisher Scientific). The DNA sequences were analyzed by DNA Dynamo software (Blue Tractor Software, North Wales, UK).

### Statistical analysis

The IC_50_ of each drug against recombinant HSVs was presented as the mean ± standard deviation. Statistical differences in IC_50_ values between WT and each of the generated recombinant viruses and among each recombinant virus were evaluated by Student’s *t*-test. The *P* value < 0.01 was considered statistically significant.

## Data Availability

The sequences of hBVTK and mBVTK are available in the GenBank database (accession no. LC764823 and AF533768.1, respectively). The sequences of DNApol gene with amino acid substitution L951R, G841S, R700G, E798K, A605V, A719T, and A719V found in ACV-resistant HSV-1_hBVTK clones are available in the GenBank database (accession no. LC782742 to LC782748, respectively).

## SUPPLEMENTAL MATERIAL

Fig. S1 spectrum.03091-23-s0001.pdf Construction of transfer DNA containing BV TK.

ASM does not own the copyrights to Supplemental Material that may be linked to, or accessed through, an article. The authors have granted ASM a non-exclusive, world-wide license to publish the Supplemental Material files. Please contact the corresponding author directly for reuse.
