# Supplementary material for: Analysis of antiviral drug properties of thymidine kinase of herpes B virus using recombinant herpes simplex virus 1
Source: Microbiol Spectr. 2023 Dec 14;12(1):e03091-23. doi: 10.1128/spectrum.03091-23 (PMC10783082; doi:10.1128/spectrum.03091-23)

Supplementary figure 1. Construction of transfer DNA containing BV TK. DNA containing 50 nucleotides of the 3'-end of cytomegalovirus (CMV) promoter, the full length hBVTK gene and its poly A tail, and a 40-nucleotide sequence of UL51 flanked by *BamHI* restriction sites was amplified from pEP-KanS_hBVTK by PCR (DNA fragment) . The DNA fragment was inserted into the vector pAmp-9mScarlet-I DNA to obtain pAmp-9mScarlet-1_hBVTK. DNA containing 50 nucleotides of the 3'-end of the CMV promoter, a *I-Sce*I site, an ampicillin resistance cassette, the mScarlet-I gene, 50 nucleotides of the 3'-end of CMV promoter , hBVTK gene and its poly(A) region, and 50 nucleotides of the HSV-1 UL51 gene was was amplified from pAmp-9mScarlet-1_hBVTK (Transfer DNA).

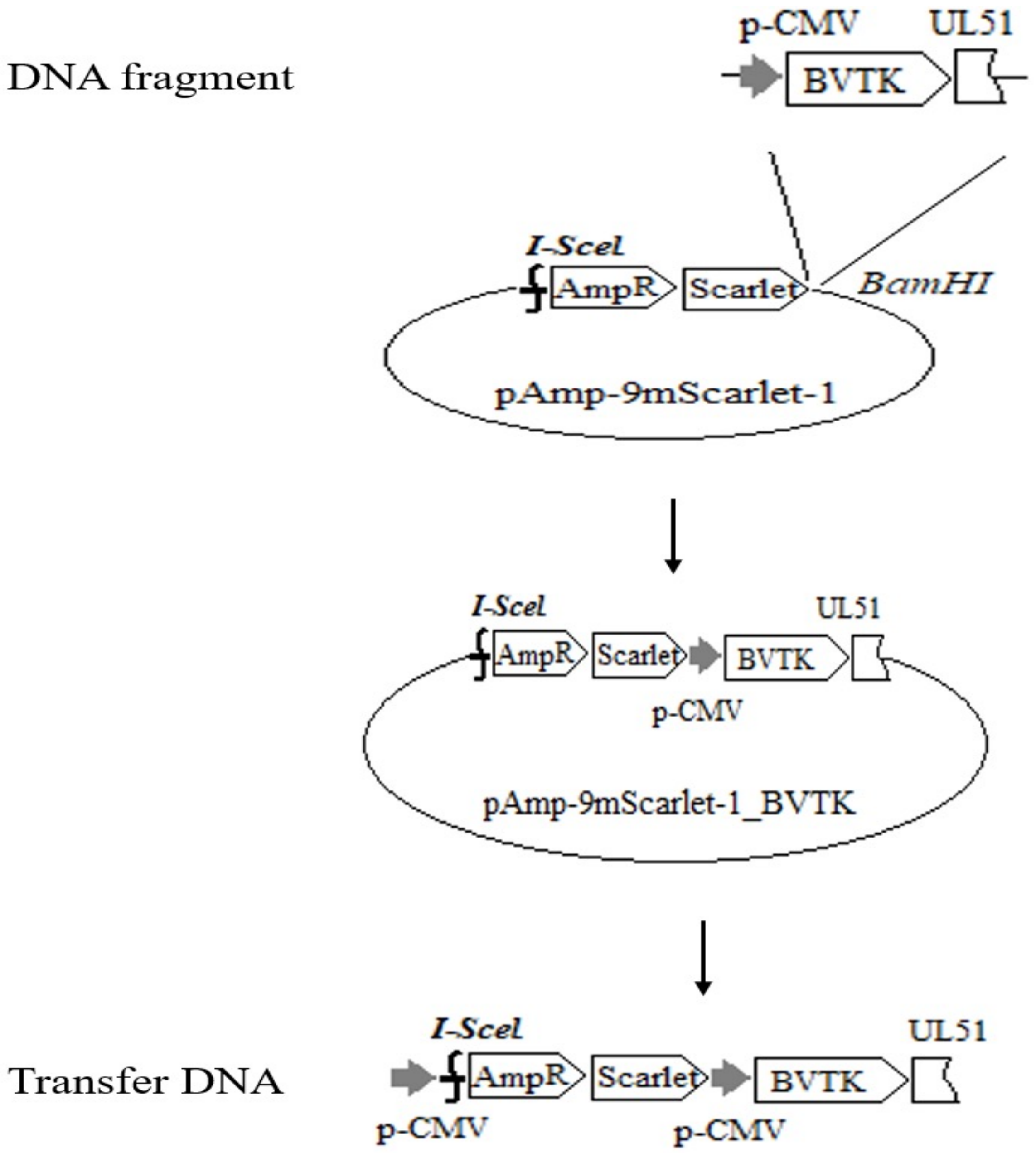

Supplement: Fig. S1 — Construction of transfer DNA containing BV TK. [file spectrum.03091-23-s0001.pdf]
